# Nicotinamide, Nicotinamide Riboside and Nicotinic Acid—Emerging Roles in Replicative and Chronological Aging in Yeast

**DOI:** 10.3390/biom10040604

**Published:** 2020-04-15

**Authors:** Ivan Orlandi, Lilia Alberghina, Marina Vai

**Affiliations:** 1 Dipartimento di Biotecnologie e Bioscienze, Università di Milano-Bicocca, 2016 Milan, Italy; ivan.orlandi@unimib.it; 2 ISBE.IT/SYSBIO Centre for Systems Biology, 2016 Milan, Italy; lilia.alberghina@unimib.it

**Keywords:** vitamin B3, NAD^+^ metabolism, Sir2, aging, yeast

## Abstract

Nicotinamide, nicotinic acid and nicotinamide riboside are vitamin B3 precursors of NAD^+^ in the human diet. NAD^+^ has a fundamental importance for cellular biology, that derives from its essential role as a cofactor of various metabolic redox reactions, as well as an obligate co-substrate for NAD^+^-consuming enzymes which are involved in many fundamental cellular processes including aging/longevity. During aging, a systemic decrease in NAD^+^ levels takes place, exposing the organism to the risk of a progressive inefficiency of those processes in which NAD^+^ is required and, consequently, contributing to the age-associated physiological/functional decline. In this context, dietary supplementation with NAD^+^ precursors is considered a promising strategy to prevent NAD^+^ decrease and attenuate in such a way several metabolic defects common to the aging process. The metabolism of NAD^+^ precursors and its impact on cell longevity have benefited greatly from studies performed in the yeast *Saccharomyces cerevisiae*, which is one of the most established model systems used to study the aging processes of both proliferating (replicative aging) and non-proliferating cells (chronological aging). In this review we summarize important aspects of the role played by nicotinamide, nicotinic acid and nicotinamide riboside in NAD^+^ metabolism and how each of these NAD^+^ precursors contribute to the different aspects that influence both replicative and chronological aging. Taken as a whole, the findings provided by the studies carried out in *S. cerevisiae* are informative for the understanding of the complex dynamic flexibility of NAD^+^ metabolism, which is essential for the maintenance of cellular fitness and for the development of dietary supplements based on NAD^+^ precursors.

## 1. Introduction

The nicotinamide adenine dinucleotide (NAD) is an essential pyridine compound that is required for cellular bioenergetics and metabolism and is also a critical regulator of key cellular processes including epigenetic modifications, DNA damage repair and aging/longevity [1,2,3]. In fact, in several pathways such as glycolysis, the TCA cycle, oxidative phosphorylation and β-oxidation, NAD is a cofactor of many redox reactions acting as acceptor/donor of electrons by the interplay between an oxidized and reduced state [1]. In addition, as NAD^+^ is a mandatory co-substrate for some NAD^+^-consuming enzymes, among which Sirtuins represent an evolutionarily conserved family of type III deacetylases [4,5,6]. Sirtuins, by coupling NAD^+^ cleavage and deacetylation of target proteins (histones and non-histone substrates), are the functional connection between the cellular energy metabolism and the regulation of aging/longevity in diverse organisms in response to a variety of nutritional and environmental stimuli [1,7,8].

Consequently, the maintenance of a proper NAD intracellular pool is essential to fuel redox metabolism and preserve the whole cellular redox state, as well as to support NAD^+^-dependent pathways. However, unlike redox reactions, which do not change the overall NAD amount, Sirtuins-mediated deacetylation and other cellular processes involving NAD^+^-consuming enzymes, affect NAD^+^ levels. Thus, a constant replenishment of NAD^+^ is postulated as required for preserving efficient cellular fitness. In this context, an age-dependent decrease in NAD^+^ content occurs in many organisms, that contributes to the development of the age-associated metabolic decline and the development of several age-related diseases [7,9,10]. In addition, it has been shown that supplementation of some NAD^+^ precursors has health benefits and can attenuate certain deficiencies associated with the aging process [11,12,13,14,15]. In this regard, a mini-review has been published [16] based on results of NAD^+^ precursor supplementation, which have been presented by basic researchers and clinicians in a recent meeting (3rd NO-Age Symposium, 2019). These NAD^+^ precursors, namely nicotinic acid (NA), nicotinamide (NAM) and nicotinamide riboside (NR), belong to the group of vitamin B3 and are used for NAD^+^ biosynthesis across species, spanning from yeast to human [1,12,17,18,19]. They are available in the diet since they are present in traces in some daily foods (vegetables, fruits, meat and milk) and are currently the object of considerable interest in the nutraceutical field, with the aim of using them as dietary supplements with preventive pro-healthy aging properties.

In this review, we refer to studies performed in the budding yeast *Saccharomyces cerevisiae*, a well-established model system that has contributed a great deal to the understanding of NAD metabolism and aging. As far as the former is concerned, due to the broad-ranging interest in NAD^+^ precursors, a particular focus is given to the role of NA, NAM and NR in the complex network of reactions that are involved in the synthesis and homeostasis of NAD^+^. In this context, we comprehensively summarize results regarding the effects of these precursors on yeast replicative and/or chronological aging. These latter are two complementary aging models. They simulate cellular aging of mitotically active cells, such as fibroblasts and that of post-mitotic quiescent cells such as myocytes, respectively [20,21,22]. Finally, possible regulatory circuits linking these NAD^+^ precursors, cellular metabolism and yeast longevity, are discussed.

Concerning NAD^+^ metabolism in mammals and its comparison with that in yeast, both have been analyzed in detail in some pertinent reviews [23,24,25,26,27].

## 2. Nicotinic Acid, Nicotinamide and Nicotinamide Riboside: Key Metabolites for NAD^+^ Homeostasis

**Figure 1:**
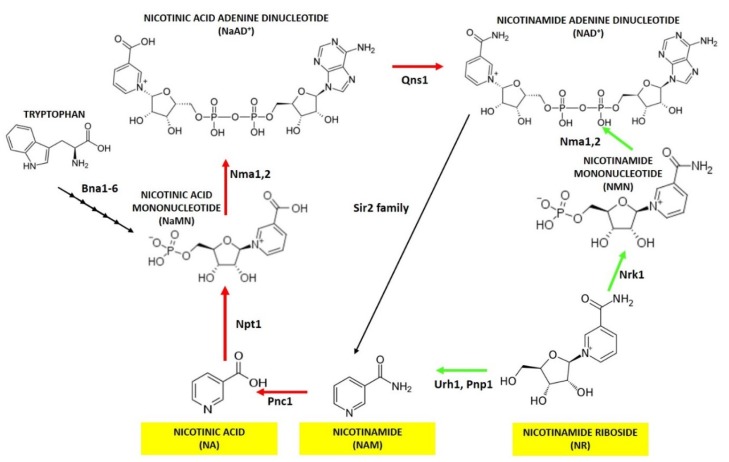
NAD^+^ synthesis in yeast. A schematic view of the pathways involved in NAD^+^ biosynthesis. In yeast NAD^+^ can be obtained through the *de novo* pathway starting from tryptophan or through *salvage* pathways from nicotinic acid (NA), nicotinamide (NAM) and nicotinamide riboside (NR) evidenced in yellow boxes. Abbreviations of enzyme names, that catalyze each step of NAD^+^ biosynthesis, are reported. The Preiss-Handler pathway is indicated by red arrows while NR utilization is indicated by green ones.

**Figure 2:**
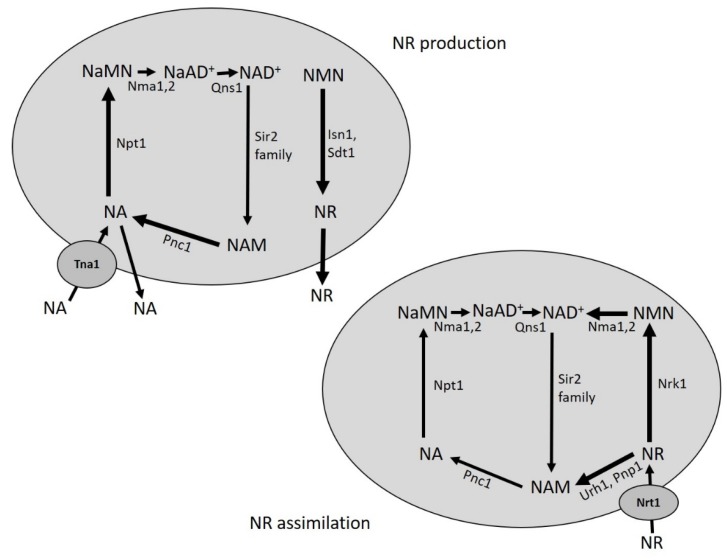
Simplified scheme of NA and NR metabolism in yeast. NA and NR are constitutively produced, released in the growth medium and retrieved by yeast cells. Uptake of NA and NR depends on Tna1 and Nrt1, respectively. NA fuels the Preiss-Handler pathway and NR enters the Nrk1-dependent and Urh1/Pnp1-dependent *salvage* routes. NA is synthesized intracellularly from NAM, while NR from NMN. Abbreviations of enzyme names are reported. See text for details.

From yeast to mammalian cells, NAD^+^ is generated either *de novo* starting from L-tryptophan or by *salvage* pathways that utilize preformed precursors, which are supplied exogenously or are intracellular pyridines also retrieved from NAD^+^-consuming reactions. NA, NAM and NR are among these precursors [1,18]. In yeast, the *de novo* synthesis, also referred to as the kynurenine pathway [18], leads to the production of nicotinic acid mononucleotide (NaMN) through six enzymatic reactions catalyzed by Bn1-6 and a spontaneous cyclization (Figure 1). NaMN is the converging point of NA/NAM *salvage* pathways. Indeed, NaMN can be obtained following the transfer of the phosphoribose moiety of phosphoribosylpyrophosphate to NA by the nicotinic acid phosphoribosyltransferase, Npt1. Then, NaMN is adenylylated by nicotinic acid mononucleotide adenyltransferases (Nma1/Nma2) producing nicotinic acid adenine dinucleotide (NaAD). Finally, NaAD is amidated by the glutamine-dependent NAD^+^ synthetase Qns1, generating NAD^+^ (Figure 1) [28]. The pathway starting from NA to NAD^+^ generation is generally known as the Preiss–Handler pathway [29]. NA can be either imported into the cells by the high affinity permease Tna1 (K_M_ for NA about 1.7 µm) [30] (Figure 2) or produced intracellularly through deamination of NAM mediated by the pyrazineamidase and nicotinamidase Pnc1 (Figure 1) [31]. Indeed, unlike mammalian cells, *S.cerevisiae* does not possess a nicotinamide phosphorybosyltransferase, the enzyme that converts NAM to nicotinamide mononucleotide (NMN). NAM is an intracellular product of Sirtuins-mediated deacetylation. Indeed, Sirtuins consume one molecule of NAD^+^ for every acetyl residue removed, generating O-acetyl-ADP ribose and a salvageable NAM that, in turn, can be used for NAD^+^ synthesis [32,33]. In *S.cerevisiae*, Sirtuins comprise five members (Sir2, Hst1-4) [9]. Since NA is commonly supplied in standard yeast growth medium, the majority of NAD^+^ synthesis relies on NA/NAM *salvage* pathways in exponentially growing yeast cells [34].

Intracellular NAM is also produced following phosphorolysis of NR catalyzed by Pnp1 and Urh1 [35], connecting NR *salvage* pathway to NA/NAM ones (Figure 1). NR, which can enter the cells by Nrt1 transporter [36], is also converted to NAD^+^ by another *salvage* route that requires the phosphorylation of NR by the nicotinamide riboside kinase Nrk1 producing NMN (Figure 1 and Figure 2) [17]. The last one can be directly converted to NAD^+^ by Nma1/Nma2, which display dual specificity towards NMN and NaMN [37] (Figure 1). Unlike NA, NR is lacking in standard yeast growth media, nevertheless, NR can replace NA to stably maintain intracellular NAD^+^ levels [35]. Moreover, mutants defective in both *de novo* and NA/NAM *salvage* pathways require NR supplementation for growth [17]. Notably, both NR and NA are also released into the extracellular medium (Figure 2) [38,39]. The export and the transporter-mediated import of these NAD^+^ precursors give rise to a bidirectional flux between intracellular and extracellular compartments that contributes to the regulation of intracellular NAD^+^ pool [38,39]. Such a dynamic flexibility may be useful to adapt NAD^+^ availability to changes in growth conditions and in response to metabolic stresses [38,39].

**Figure 3:**
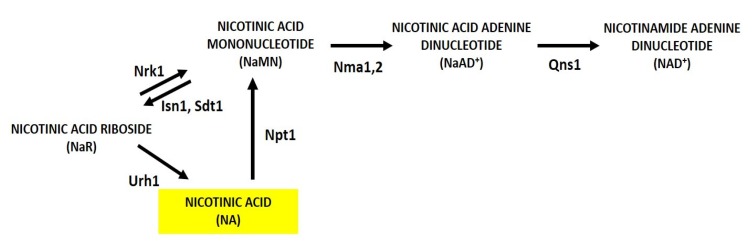
Scheme of NaR metabolism in yeast. NaR can be utilized for NAD^+^ synthesis through two *salvage* routes that rely on Nrk1 and Urh1 and produce NaMN and NA respectively. NaR is generated intracellularly from NaMN by Isn1 and Sdt1. Abbreviations of enzyme names are reported.

Finally, nicotinic acid riboside (NaR) is another nucleoside that can be utilized as an NAD+ precursor through Nrk1-dependent and Nrk1- independent pathways [40,41]. In the former, NaR is salvaged by Nrk1 producing NaMN and in the latter Urh1 is responsible for the majority of NaR utilization producing NA (Figure 3) [41]. NaR is generated intracellularly from NaMN by the nucleotidases Isn1 and Sdt1 (Figure 3) [42], which are also involved in NR synthesis (Figure 2 and see Section 3.2). Differently from NR, NaR is a poor supplement and its import into the cells depends on methyl ester modification [41].

## 3. NAD^+^ Precursors and RLS

In the next sections, we summarize and analyze the effects of NA, NAM and NR on yeast replicative aging. Due to the asymmetrical cellular division (budding) of *S.cerevisiae*, replicative aging is mother cell-specific and is characterized by the limited capacity of a mother to produce daughter (bud) cells. The number of buds that a mother can generate in the presence of nutrients before she dies allows us to measure the replicative potential or replicative lifespan (RLS) [43]. In the context of replicative aging, among NAD^+^-consuming enzymes, the founding member of the Sirtuin family, namely Sir2, plays a major role as an anti-aging factor. Indeed, Sir2 deacetylase activity promotes RLS [44,45]. Sir2 is required for gene silencing at telomeres, mating-type loci (*HML* and *HMR)* and rDNA *locus*, where it is responsible for establishing and maintaining a hypoacetylated chromatin state [46]. *SIR2* loss of function results in RLS decrease in concert with a reduction in silencing and increased recombination at the rDNA *locus* [33,47]. Such an increased recombination has been reported during replicative aging and contributes to genomic instability, a factor that negatively affects RLS [48,49,50]. Furthermore, an age-related increase of H4 lysine 16 acetylation occurs in specific subtelomeric regions related to the lack of Sir2-targeted deacetylation in replicatively old cells, which results in reduced silencing [51]. Consequently, in assessing the effects of NAD^+^ precursors on replicative longevity, much attention has been paid to Sir2 silencing activity.

### 3.1. NA, an NAD^+^ Precursor Influencing RLS

**Figure 4:**
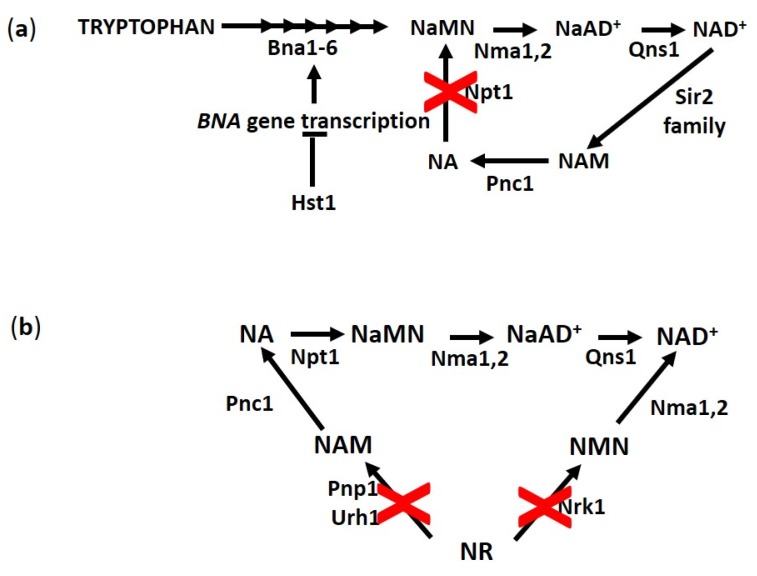
Scheme of main biosynthetic routes for NAD^+^. (**a**) Hst1 negatively controls the transcription of *BNA* genes required for *de novo* NAD^+^ synthesis in yeast. The inactivation of *NPT1* prevents NA and NAM recycling through *salvage* pathways. (**b**) Cells lacking Nrk1, Pnp1 and Urh1 cannot utilize NR for NAD^+^ synthesis. Abbreviations of enzyme names are reported.

NA, as stated previously, is present in synthetic yeast media and is imported efficiently into the cells by Tna1. However, NA is not an essential supplement for cells with a functional *de novo* pathway. Indeed, cells grow well in NA-free synthetic medium but display a reduced RLS and poor telomeric silencing [35]. Since in this growth condition the intracellular NAD^+^ concentration is about 1 mM, while in the synthetic medium containing NA is about 2 mM [35], it has been suggested that 1 mM NAD^+^ allows cells to cope with metabolic requirements for growth but is limiting for Sir2 activity [35]. This hypothesis has been proposed considering NAD^+^ K_M_ of Sir2, which is 29 µM [52]. Although this affinity could be satisfied by an intracellular concentration of 1 mM NAD^+^, one must take into account, on the one hand, the NAD^+^ subcellular compartmentalization (mitochondria, cytosol, nucleus) and, on the other, that the majority of NAD^+^ is protein-bound [53]. Thus, the amount of free nuclear NAD^+^ can be below the adequate co-substrate requirement for Sir2 activity. The presence of NA in the growth medium and, consequently, the availability of precursors for the *salvage* pathway increases NAD^+^ levels. Such an increase promotes Sir2 functions and extends RLS [54]. In line with this, cells lacking the NA *salvage* enzyme Npt1 are characterized by normal growth, severe decrease in NAD^+^ levels, loss of Sir2- mediated silencing and a short RLS [35,55,56,57]. In addition, *npt1* null mutants display upregulation of genes of the *de novo* pathway, while those of the *salvage* pathway are unaffected indicating that these null mutants, being defective in NA/NAM *salvage*, rely on the *de novo* pathway for NAD^+^ biosynthesis [58]. This pathway can satisfy the requirements essential for growth but not those for Sir2 functions affecting negatively RLS. Interestingly, the transcription of genes of *de novo* pathway are under the control of Hst1 (Figure 4a). It has been proposed that this NAD^+^-dependent deacetylase can also act as an NAD^+^ sensor and regulator of NAD^+^ levels adjusting the degree of *BNA* gene repression [58] In *npt1* null mutants, a feedback mechanism takes place where the low NAD^+^ level decreases Hst1 activity and leads to de-repression of *BNA* genes [58]. The low NAD^+^ binding affinity of Hst1 (K_M_ of 94.2 µM) assures that *de novo* synthesis occurs and restores adequate NAD^+^ levels to fulfill growth requirement. The presence of this additional regulatory circuit controlling NAD^+^ homeostasis via Hst1 may contribute to explain why *NPT1* overexpression promotes Sir2-dependent RLS extension without increasing NAD^+^ levels [56]. 

### 3.2. NR, a New Vitamin Linking NAD^+^ Homeostasis and Longevity

NR has been shown to be an efficient NAD^+^ precursor, the recycling of which is important for NAD^+^ homeostasis, as well as for RLS [17,35,42]. NR is assimilated through the Nrk1-dependent and Urh1/Pnp1-mediated routes (Figure 2); these *salvage* routes are responsible for the utilization of exogenous NR and of that physiologically generated from NMN by the nucleotidase activities of Isn1 and Sdt1 in the cytosol (Figure 2) [38,42]. In addition, NR is also produced in the vacuole from NMN by Pho8 supporting the existence of an NR cellular compartmentalization in cytosolic and vacuolar pools. In this context, the transporter Fun26 has been suggested to have a balancing function between the two NR pools [59,60]. This might enable, on the one hand, transportation of the NR generated in the vacuole into the cytoplasm supporting NAD^+^ synthesis when needed and, on the other, that of cytosolic NR in excess into the vacuole. Thus, the regulation of assimilation, release and re-uptake of NR relies on an extended flexible NAD^+^ pool that encompasses both extracellular and intracellular compartments. Moreover, the balance between the activities of NMN adenylyltransferases and NMN nucleotidases seems to be a critical point to drive pyridine nucleotide metabolism “forward” to dinucleotides or “backward” to nucleosides depending on specific NAD^+^ requirements [60].

In such a dynamic flexibility of homeostatic mechanisms regulating NAD^+^ levels, NR supplementation to *npt1* mutants rescues Sir2-dependent silencing defects and extends RLS [35]. Similarly, NR supplementation to cells grown in NA-free synthetic medium is sufficient for suppressing Sir2-dependent silencing defects and extends RLS. This effect is dependent on NR *salvage* routes and is accompanied by an increase (from about 1 mM to about 2 mM) in intracellular NAD^+^ amount [35] that, as discussed above for NA supply, may provide support for an adequate Sir2 activity. However, although both NA and NR can boost NAD^+^ levels, the latter vitamin displays a more long-lasting effect. Indeed, following NA supplementation NAD^+^ amount decreases over the course of the culture growth, while after NR supplementation it remains stable [35]. This indicates that NR assimilation is able to prevent NAD^+^ reduction that physiologically takes place as cells age [61,62].

### 3.3. NAM: A Problem or Resource for Replicative Aging?

NAM is a salvageable vitamin that can be used as a substrate for NAD^+^ synthesis following its conversion to NA by Pnc1. It can be generated by Urh1/Pnp1-mediated catabolism of NR and as a by-product of Sir2 deacetylase reaction (Figure 4a). Moreover, NAM is also an endogenous non-competitive inhibitor of Sir2, shifting the enzymatic reaction toward the reformation of NAD^+^ and acetylated target (Figure 4a) [5,33,47]. Consequently, intracellular NAM levels are the results of the balance between its generation and consumption in the different cellular compartments. NAM supplementation (5 mM) to yeast growth media inhibits Sir2 activity resulting in a phenocopy of *SIR2* inactivation—NAM-treated cells display a reduction in silencing, an increased recombination at the rDNA *locus* and a short RLS similar to that of a *sir2* null mutant [47]. Pnc1 overexpression abrogates the inhibitory silencing effects of exogenously added NAM by converting the excess of NAM into NA and increases RLS [63]. This suggests that exogenously imported NAM, creates a high local concentration of NAM, which, if not cleared by Pnc1 in the nucleus by inhibiting Sir2, has a pro-replicative aging effect. Of note, Pnc1 overexpression alone (five additional copies) is sufficient to greatly enhance RLS in a Sir2-dependent manner [64].

In addition, both Pnc1 and Sir2 are required for RLS extension observed in calorie-restricted cells [64,65]. Although, a Sir2-independent aging pathway responsive to CR has been described [66,67]. Calorie restriction (CR) is a practice of limiting nutrient intake without malnutrition known to extend lifespan in a wide spectrum of organisms, ranging from yeast to primates [68,69,70]. In yeast, CR is generally imposed by reducing the glucose concentration in the growth medium from 2% to 0.5-0.2%: a growth condition that sustains growth as well as 2% glucose [71,72]. In this context, much work has been done on potential mechanisms by which glucose levels may affect Sir2 enzymatic activity. Different models have been proposed including, among others, that CR works in increasing NAD^+^ levels or altering the ratios of NAD^+^ metabolites [64,73] but a clear consensus has not yet been reached.

Quantification of NAD^+^ metabolites during CR indicates that glucose restriction does not affect levels or ratios of NAD^+^, NAM and NADH in such a way to be the driver of increased Sir2 activity [74]. This cannot exclude the possibility that in different intracellular compartments local changes in NAD^+^/NADH levels and in the other NAD^+^ intermediates occur. Unfortunately, to date, it is not possible to rigorously evaluate such a possibility due to the difficulty of determining the absolute levels of free pyridine nucleotides in the different compartments. However, determination of the protein copy number of the enzymes involved in NAD^+^ metabolism indicates that both Sir2 and Pnc1 are up-regulated in glucose-restricted rich media, only Pnc1 in glucose–restricted synthetic media, while the levels of the other enzymes are unchanged [75]. In addition, the increased expression of Pnc1 induced by CR correlates with increased rates of NAM hydrolysis [64] indicative of an increased flux along the *salvage* pathway.

The important contribution of NAM and of the corresponding *salvage* pathway on Sir2 activity, as well as, on RLS also emerges from experiments performed with an isostere of NAM, namely isonicotinamide (INAM), which is an NAM antagonist and a Sir2 agonist in vitro and in vivo. It activates Sir2 by relieving NAM inhibition and enhances Sir2-mediated silencing [61,76]. Moreover, it has been shown that in NA-free synthetic medium, INAM supplementation increases Sir2 activity by a combined contribution due to a relief of NAM inhibition and an enhancement of NAD^+^ amount resulting in RLS extension [61]. INAM supplementation restores the intracellular NAD^+^ concentration to levels similar to those determined in the presence of exogenous NA or NR [35], (see also NA and NR sections above). The INAM-induced increase in NAD^+^ requires Npt1 and Pnc1 consistent with an increased flux along the *salvage* pathway [61]. Such an increased flux is also fueled by NAM provided by the Urh1/Pnp1-mediated branch of NR *salvage* pathway [61].

Overall, although NAM is a powerful inhibitor of Sir2 when its intracellular concentration is high enough, as in the case of an exogenous supplementation, it is at the same time an effective precursor of NAD^+^ ensuring an adequate biological activity of Sir2, thus supporting its pro-replicative longevity role.

## 4. NAD^+^ Precursors and CLS

In the next section we analyze the impact of NAD^+^ precursors on chronological aging. Yeast cells age chronologically in a non-dividing quiescent state (stationary phase) due to nutrient depletion. The chronological lifespan (CLS) refers to the rate of post-mitotic survival of the quiescent culture. Starting 72 h after the diauxic shift, CLS is estimated by the percentage of cells able to resume growth and form a colony upon return to fresh rich medium [77]. Due to progressive changes in the nutritional availability, chronological aging is characterized by a huge metabolic reconfiguration: in particular, at the diauxic shift, when upon glucose exhaustion, cells shift from glucose-driven fermentation to ethanol/acetate-driven respiration. This reconfiguration allows cells to acquire specific features that are required for survival and principally involves an increase in mitochondrial respiration and activation of gluconeogenesis. The former is essential for chronological longevity [78,79,80], the latter supports the production of trehalose, the accumulation of which is beneficial for CLS extension [79,81]. Referring to gluconeogenesis, the first irreversible reaction is catalyzed by phosphoenolpyruvate carboxykinase (Pck1). Pck1 enzymatic activity is regulated by its de/acetylation state: an increase in the acetylated (active) form enhances gluconeogenesis and extends CLS [62,81,82,83]. The enzyme responsible for Pck1 deacetylation is Sir2 [81,82]. Consequently, during chronological aging, the negative control of Sir2 activity on the gluconeogenic activity of Pck1 leads to a negative effect also on CLS [83]. Indeed, unlike RLS, Sir2 does not promote CLS [62,81,84,85].

Thus, the possible effects of NAD^+^ precursors on chronological aging can involve, on the one hand, the mechanisms that face the physiological reduction of NAD^+^ levels observed as cells age [35,86] and, on the other, the metabolic pathways that affect CLS.

### Impact of NA, NR and NAM on CLS

Although NA, NR, NAM and their *salvage* pathways have been mainly investigated for the roles that they play in RLS regulation, as above discussed, some aspects of their involvement in chronological aging are emerging. In fact, the *nrk1Δurh1Δpnp1Δ* mutant, completely impaired in NR utilization (Figure 4b), exhibits a significantly reduced CLS. On the contrary, lack of Npt1 that prevents NAD^+^ synthesis through the NA *salvage* pathway (Figure 4a), does not affect CLS [38] differently to what has been observed for RLS [35,61]. In addition, NA supplementation to the short-lived *nrk1Δurh1Δpnp1Δ* mutant has no effect on its CLS [38]. This suggests that the NR *salvage* pathway is more critical than the NA one for CLS and that NR assimilation might be required for cell survival in stationary phase. Consistently, NR is mainly produced during late exponential phase of growth [38]. Furthermore, the NR *salvage* pathway seems to be also important for CR-induced CLS extension. Indeed, a CR regimen can increase RLS, as well as CLS [22]. The short-lived phenotype of the *nrk1Δurh1Δpnp1Δ* mutant is unaffected by CR [38].

The establishment of a quiescent program in chronologically aging cells also involves the activation of endogenous defense mechanisms that contribute to ensure long-term survival. In line with this, CLS extension of cells grown under CR is accompanied by an enhanced heat stress resistance [87,88]. In the *nrk1Δurh1Δpnp1Δ* mutant the CR-induced heat stress resistance is abolished [38].

As far as NAM is concerned, recently we reported that its supplementation at the onset of chronological aging (diauxic shift) determines CLS extension by inhibiting Sir2 activity [62]. NAM-supplemented cells phenocopy chronologically aging *sir2Δ* cells. Both these cells display the same metabolic changes which ensure a longer CLS. In particular, lack of Sir2 or NAM-mediated Sir2 inhibition correlate with an increase in the acetylated active form of Pck1 due to the lack of Sir2-targeted deacetylation [62,81,82]. This results in an enhanced gluconeogenesis in concert with an increased accumulation of trehalose. In addition, NAM-mediated Sir2 inhibition, as well as *SIR2* inactivation, are associated with a more efficient respiratory activity, characterized by a reduced non-phosphorylating respiration that in turn correlates with a decreased burden of harmful superoxide anion [62]. Taken together, all these features favor a better long-term survival [79,80]. Moreover, lack of Sir2 further exacerbates CLS extension obtained in a severe form of CR, namely when postdiauxic cells are transferred from the expired medium to water [81,84]. NAM-supplemented cells also phenocopy the CLS of *sir2Δ* cells in such an extreme CR condition [62]. This CLS-extending effect takes place in concert with an increase of the acetylated Pck1 level [83], further supporting the importance of Sir2 activity on its cellular target Pck1 during chronological aging.

Notably, it has also been reported that the lack of Sir2 enhances the pyridine nucleotide flow into NR branch leading to an increased release of NR [38]. Since NR assimilation seems be required for cell survival during chronological aging, future studies are needed to elucidate the relationship between NR metabolism and the metabolic outcomes due to *SIR2* inactivation that extend CLS.

## 5. Production of NR from Yeast Cultures

NR has been qualified as Generally Recognized as Safe (GRAS) by the Food and Drug Administration (FDA) in the United States and by the European Food Safety Authority [89]. NR has become available as a supplement with the brand name NIAGEN (Chromadex Incorporated, Irvine, California, USA). Since *S.cerevisiae* is an organism GRAS, it could offer a great opportunity to obtain NR through a safe fermentative process, avoiding other more expensive methods of synthesis. In this context, starting from the mutant *nrk1Δurh1Δpnp1Δ*, which displays an increased NR export into the culture medium [38,42], the subsequent inactivation of *NRT1* encoding the NR transporter results in a strain accumulating in the medium about 4.06 ± 0.9 µM of NR against 0.12 ± 4 µM of the wild type [39]. Further optimization of growth and media composition, including NA supplementation, leads to a further increase of extracellular NR level reaching 28.2 ± 8.5 µM that was purified by a two-step process (solubilization in cold methanol and separation on SP-sephadex column) [39]. On the whole, these results suggest the possibility to utilize cheaper vitamins (NA) to obtain NR as a product that can be easily recovered in a purified form.

## 6. Conclusions

NAD^+^ metabolism is tightly regulated as well as highly dynamic and flexible. These features result from the integration of the different NAD^+^ biosynthetic pathways (*de novo* and *salvage* routes) in a complex network at the level of some key intermediates. This prevents NAD^+^ imbalance and ensures cells to cope with metabolic requirements according to changes in environmental/physiological conditions including aging. Indeed, during aging a systemic decline of NAD^+^ occurs that contributes to reduce the fitness of the organism over time [9,10]. NA, NAM and NR, NAD^+^ precursors spanning from yeast to humans, fulfil such critical functions in supporting NAD^+^ metabolism/homeostasis and directly affect both replicative and chronological yeast aging, as evidenced by various aspects here presented.

In particular, an interesting aspect that emerges from investigating the role of NA and NR is the existence of a compartmentalization of these NAD^+^ precursors in intracellular and extracellular pools [56,59], which confer the metabolic flexibility required for supporting the actual demands of cells. In addition, it cannot be ruled out the possibility that the compartmentalization might be a further means to regulate intracellular enzymatic activity. In fact, along the Pnc1-mediated route salvageable NAM is converted into NA, which can be secreted [39]. Since NAM is an endogenous non-competitive inhibitor of Sir2 [5,33], NA export can allow on the one hand, an adequate flux through the NA *salvage* pathway and, on the other, a fine control of the deacetylase activity of this Sirtuin, which is a well-known modulator of aging. In this context, when exogenous NAM is provided to the cells at a concentration sufficient to inhibit Sir2, two opposite outcomes are obtained according to the opposite role played by Sir2 in replicative and chronological aging. In the former, the Sir2 inhibition results in a short RLS, while in the latter in a CLS extension [62,81,84].

Concerning NA and NR, NR assimilation, in particular, appears to be more effective in promoting the survival of yeast cells in both replicative and chronological aging. Furthermore, NR has been recognized as GRAS and is orally bioavailable as a dietary supplement (NIAGEN). This has provided the opportunity to assess the potential benefits of NR observed in laboratory animal models to humans. Promising results have been obtained from preclinical studies [14,90,91] and some clinical trials have determined or are currently determining safety, tolerance and efficacy of NR supplementation in adults [11]. Published clinical data indicate that NR is safe and well-tolerated [92,93,94,95]. However, since in men with obesity no improvement has been observed following NR supplementation on some impaired physiological functions associated with obesity (glucose tolerance, mitochondrial functionality) [95,96,97], larger-scale clinical trials are required that may include women and individuals with other diseases.

Finally, taking into account the information obtained in yeast on the different contributions of NAM, NA and NR on replicative and chronological aging, in perspective one could hypothesize a differentiated use of them in order to achieve health benefits. In particular NAM and NR could offer a promising strategy for postmitotic cells while NA and NR for actively dividing cells. Further studies are required to understand whether these NAD^+^ precursors can have beneficial or detrimental effects depending on cell type and tissue in humans.
